# Characteristics and Rates of Preterm Births During the COVID-19 Pandemic in Germany

**DOI:** 10.1001/jamanetworkopen.2024.32438

**Published:** 2024-09-10

**Authors:** Birte Staude, Björn Misselwitz, Frank Louwen, Ulrich Rochwalsky, Frank Oehmke, Siegmund Köhler, Rolf F. Maier, Anita C. Windhorst, Harald Ehrhardt

**Affiliations:** 1 Department of General Pediatrics and Neonatology, Universities of Giessen and Marburg Lung Center, Member of the German Center for Lung Research, Justus-Liebig-University Giessen, Giessen, Germany; 2 Federal State Consortium of Quality Assurance Hesse, Eschborn, Germany; 3 Division Obstetrics and Prenatal Medicine, Goethe University Frankfurt–Main, Frankfurt, Germany; 4 Division of Neonatology, University of Frankfurt, Frankfurt, Germany; 5 Department of Gynecology and Obstetrics, Justus Liebig University of Giessen, Giessen, Germany; 6 Center of Obstetrics and Gynecology, University of Marburg, Marburg, Germany; 7 Children’s Hospital, University Hospital, Philipps University Marburg, Marburg, Germany; 8 Institute of Medical Informatics, Justus-Liebig-University Giessen, Giessen, Germany; 9 Division of Neonatology and Pediatric Intensive Care Medicine, Department of Pediatrics and Adolescent Medicine, University Medical Center Ulm, Ulm, Germany

## Abstract

**Importance:**

Population-based analyses provided divergent data on the changes in preterm birth rates during the COVID-19 pandemic, and there is a gap of knowledge on the variations in birth characteristics.

**Objective:**

To study changes in perinatal care, causes of preterm delivery, and very preterm (VPT; defined as <32 weeks’ gestation) birth rates before and during the COVID-19 pandemic.

**Design, Setting, and Participants:**

This population-level cohort study used data from the quality assurance registry, which covers all births in Hesse, Germany. Deliveries during the COVID-19 pandemic (2020) were compared with the corresponding grouped prepandemic time intervals (2017 to 2019). Analyses were executed between August 2023 and July 2024.

**Exposures:**

Analyses were directed to study differences in preterm births before and during 3 pandemic phases: first (March 14 to May 15, 2020) and second (October 19 to December 31, 2020) lockdowns and a period of less-vigorous restrictions between them (May 16 to October 18, 2020).

**Main Outcomes and Measures:**

Outcomes of interest were variations in preterm birth rates in the context of baseline characteristics and causes of preterm births during vs before the first year of the COVID-19 pandemic.

**Results:**

From the total cohort of 184 827 births from 2017 to 2020, 719 stillbirths occurred and 184 108 infants were liveborn. Compared with the prepandemic period, medical care characteristics did not differ during the COVID-19 period. The odds of VPT births were lower during the pandemic period (odds ratio [OR], 0.87; 95% CI, 0.79-0.95) compared with the prepandemic period, with the greatest reduction observed during the second lockdown period (OR, 0.69; 95% CI, 0.55-0.84). Reduction in VPT births was attributed to fewer births in pregnancies among individuals with a history of serious disease (OR, 0.64; 95% CI, 0.50-0.83), pathologic cardiotocography (OR, 0.66; 95% CI, 0.53-0.82), and intrauterine infection (OR, 0.82; 95% CI, 0.72-0.92) while incidences of history of preterm birth, multiple pregnancies, serious or severe psychological distress, and preeclampsia, eclampsia, or hemolysis, elevated liver enzymes, and low platelet count syndrome as cause for preterm delivery remained unchanged.

**Conclusions and Relevance:**

In this population-based cohort study on the COVID-19 pandemic and preterm birth rates, the duration of exposure to mitigation measures during pregnancy was associated with accelerated reductions in preterm births. The findings of lower rates of baseline risks and causes of preterm deliveries support efforts to intensify health care prevention programs during pregnancy to reduce the preterm birth burden. These findings of this study put particular focus on hygiene measures to reduce the rate of deliveries for intrauterine infection and highlight the potential of expanding strategies to the different risks and causes of preterm delivery.

## Key Points

**Question:**

Were there changes in preventable risk factors, causes of preterm birth, and delivery rates during the 3 COVID-19 pandemic phases in Germany in 2020 compared with previous years?

**Findings:**

In this cohort study including all 184 827 births in Hesse, Germany, from 2017 through 2020, reduction of preterm births increased with the ongoing lockdown measures. While quality markers of prenatal care remained unchanged, decreases were observed in high-risk pregnancies with history of maternal serious disease and deliveries for pathologic cardiotocography and intrauterine infection.

**Meaning:**

The findings of this cohort study highlight opportunities for prevention measure programs intended to reduce potentially modifiable risks for preterm delivery and to intensify financial and research investments to reduce preterm deliveries.

## Teaser

This cohort study assesses changes in perinatal care, causes of preterm delivery, and rates of preterm delivery before and during the COVID-19 pandemic in Hesse, Germany.

## Introduction

The COVID-19 pandemic resulted in dramatic restrictions for daily life. The lockdown measures incidentally changed the access to health care services, as well as personal attitudes to use of medical care.^1,2^ The change in perception of health-related risks for pregnant individuals might have impacted contact behavior among the individuals in general.^3,4,5,6^ Several studies have evaluated the associations between the onset of the COVID-19 pandemic and preterm birth rates based on patient registries and found diverse results, with unchanged or reduced preterm birth rates. While early reports from Denmark and Ireland observed dramatic reductions, no decline was found for Norway, Sweden, or Denmark when the observation period was expanded beyond the initial lockdown period.^7,8,9,10,11^ Two systematic meta-analyses concluded that no overall reduction in preterm birth rates occurred during the COVID-19 pandemic, but any effects might have been restricted to areas with more restrictive lockdown measures and high-income countries.^12,13^ The decline in preterm birth rates in the US with the 2020 lockdown was attributed to decreases in cesarean deliveries and induced deliveries, while pregnancy risk-factors of self-pay status, history of premature delivery, and maternal diabetes or arterial hypertension were associated with an increased risk of preterm birth in Colorado.^14,15^

Reports on changes in stillbirth rates provided divergent results, and most analyses did not consider the potential trade-offs between stillbirth and preterm birth rates.^2,6,10,16,17,18,19,20,21,22^ A more detailed analysis from Australia detected fewer deliveries for fetal compromise but an increase in stillbirths.^22^

While most studies focused on maternal and neonatal outcomes, few studies detailed the associations of the COVID-19 pandemic with changes in maternal baseline characteristics, use of routine health care services during pregnancy, perinatal care in impeding preterm delivery, and causes of preterm birth.^3,4,12,22^ These aspects were addressed as objectives of our population-level descriptive epidemiological cohort study in the federal state of Hesse, Germany, with more than 6.2 million inhabitants and more than 60 000 births annually between 2017 and 2020.

## Methods

This cohort study was approved by the ethics committee of the Justus-Liebig-University Giessen. The requirement for informed consent was waived for the retrospective analysis of anonymous data. We adhered to the Strengthening the Reporting of Observational Studies in Epidemiology (STROBE) reporting guideline.

### Data Source and Study Population

The quality assurance registry of Hesse constitutes an obligatory medical reporting system for all deliveries of stillbirths at 500 g or more and liveborn infants with at least 22 0/7 weeks’ gestation in the state. Deliveries are recorded within the documentation system in obstetric and neonatal departments from patient records after discharge by the medical staff of each center based on predefined definitions of the database, which partly differ from *International Statistical Classification of Diseases and Related Health Problems, Tenth Revision *(*ICD-10*) codes. Data were reviewed for plausibility during data entry and export. Assessments of completeness of datasets were executed by the quality assurance body based on the accounting data of the hospitals and were consistently greater than 99%. All items remained unchanged during the study period and were prospectively collected into the database before start of the analyses.

The analyses were directed toward very preterm (VPT) infants delivered at less than 32 weeks’ gestation, and results were put into the context of maternal baseline characteristics and causes of VPT birth. Additionally, outcomes were reassessed among the more mature infants born between 32 0/7 and 36 6/7 weeks. COVID-19 pandemic restrictions in Hesse became effective on March 14, 2020. Due to the reported seasonal variations in preterm birth rates, the observation periods from January 1 to March 13 were excluded from the datasets in the COVID-19 and control collectives.^23^ The COVID-19 pandemic was further separated into 3 phases: the first lockdown (March 14 to May 15, 2020), a period of less vigorous restrictions (May 16 to October 18, 2020), and the second lockdown (October 19 to December 31, 2020) that differed from the first lockdown mainly by the continued personal school attendance across all educational levels but comparable restrictions at the workplace and in private life. Data were compared with the grouped prepandemic corresponding time intervals during 2017 to 2019 (eFigure 1 and eFigure 2 in Supplement 1).

### Definition of Outcomes and Covariates

Our primary outcomes were preterm birth rates before compared with after the onset of the COVID-19 pandemic and changes during the different phases of the pandemic. Maternal baseline characteristics included maternal age, history of maternal serious disease or family history of serious disease, history of preterm birth, history of previous cesarean delivery or uterine surgery, complications during previous pregnancies, and singleton or multiple pregnancy. Pregnancy risks included diabetes during pregnancy, maternal obesity, serious or severe psychological burden during pregnancy, preexisting hypertension, bleeding at less than 28 weeks’ gestation or at 28 weeks’ gestation or greater, and placenta previa. Prenatal care items included the number of routine medical check-ups during pregnancy, gestational age at admission, period between hospital admission and delivery, antenatal steroid (ANS) application, and mode of delivery as spontaneous birth or cesarean delivery, with separate analysis of emergency cesarean delivery and stillbirth. ANS administration was counted when at least 1 dose was given, irrespective of the interval to delivery.

Categories for preterm deliveries were separated into spontaneous onset with preterm labor, amniotic infection, and premature rupture of membranes, which were summarized under the term delivery for intrauterine infection that fall into the category of nonindicated deliveries; indicated births included preeclampsia, eclampsia, hemolysis, elevated liver enzymes, low platelet count (HELLP) syndrome, placental insufficiency with intrauterine growth restriction, placental abruption, and pathologic cardiotocography (CTG). Intrauterine infection was counted when at least 1 clinical criterion was fulfilled. Premature rupture of membranes was defined as occurring more than 18 hours before onset of labor.

Gestational age was determined by routine obstetric assessment based on the date of the last menstrual period and routine ultrasonographic measurements. Further items included sex, multiple births, pH and base excess from the arterial umbilical cord blood, and Apgar scores at 1, 5, and 10 minutes of life.

### Statistical Analysis

All statistical analyses were performed with R software package version 4.2.2 (R Project for Statistical Computing). The study used exploratory and descriptive statistical methods. As our retrospective descriptive epidemiological study was not driven by prespecified hypotheses, no predefined cutoff *P* value was used to indicate statistical significance; instead, calculated *P* values are reported.

Birth rates were presented as absolute and relative frequencies for all births, segmented by gestational age at birth and lockdown phase. Odds ratios (OR) with 95% CIs were calculated, with CIs based on Fisher exact test. Contingencies between birth rate and birth year were assessed using the Pearson χ^2^ test or Fisher exact test for variables with a small number of cases. Two logistic regression models were analyzed to assess the association between birth year and the incidence of VPT births. First, the trend variable was calculated with year as numeric variable but without 2020, then the trend was calculated with 2020. Models were compared using Akaike Information Criterion. Contingency tables were created to compare the counts of premature and term births in each year category. Fisher exact test was used to determine the statistical significance of differences. Furthermore, the distribution of indicated and nonindicated deliveries was studied in 2020 compared with 2017 to 2019.

Maternal baseline and pregnancy characteristics, along with neonatal characteristics, were described using absolute and relative frequencies. Qualitative variables, like mode of delivery or sex, were analyzed using the Pearson χ^2^ test for contingency tables, and quantitative variables were summarized using medians and IQRs, using the 2-sided Wilcoxon rank-sum test for 2-sided hypotheses.

For pregnancy and birth risks, which involve multiple answer sets and result in a large number of parameters tested, *P* values were adjusted for multiple testing using the Holm method.^24^ In addition to ORs, Fisher exact test *P* values, 95% CIs, and adjusted *P* values were reported.

To investigate the association of the lockdown with VPT births, logistic regression models were constructed. Stratified models were included as an exploratory analysis to account for potential confounding factors (eg, maternal age >35 years, pathological CTG, and the occurrence of either amniotic infection syndrome, preterm labor, or premature rupture of membranes). Models were compared using Akaike Information Criterion, and the Tjur pseudo *R*^2^.^25^ Analyses were executed between August 2023 and July 2024.

## Results

### Live Births, Preterm Births, and Stillbirths Before and During the COVID-19 Pandemic

Our analyses sample covered 184 827 births overall from 2017 to 2020, including 719 stillbirths and 184 108 liveborn infants. A total of 901 infants were not considered in analyses because the gestational age at birth had not been documented. Pandemic-era enrollment was restricted to 44 481 births from March 14 to December 31, 2020, when lockdown restrictions were effective, and this period was further separated into the first (9207 births) and second (10 204 births) lockdowns and a period between them with less vigorous restrictions (25 070 births) (eFigure 1 in Supplement 1). Further details on the numbers of births are detailed within the flowchart (eFigure 2 in Supplement 1).

There was a 4.9% reduction in overall live births in 2020 compared with the prepandemic period (Table 1). There was also a lower rate of VPT births during the COVID-19 pandemic, with 572 preterm births (1.29%) documented in 2020, compared with 2064 preterm births (1.47%) during the control periods (Table 1). Odds of preterm birth were significantly lower during 2020 compared with the control period (OR, 0.87; 95% CI, 0.79-0.95) (Table 2; eFigure 3 in Supplement 1). We included a time-series analysis of the rates of total births and VPT births during 2017 to 2019 and 2017 to 2020 and observed an accelerated decrease of VPT births if 2020 was included (eTable 1 in Supplement 1). Analysis segregating VPT births into the different phases of the COVID-19 pandemic in 2020 found that the most prominent reduction in VPT births occurred in the third period, which corresponded with an equivalent reduction in total births (OR, 0.69; 95% CI, 0.55-0.85) (Table 2). Comparable changes were observed in the analyses of all preterm infants born at less than 37 weeks’ gestation and when considering preterm infants born 32 0/7 to 36 6/7 weeks (Table 1; eTable 2 in Supplement 1). Analysis of stillbirth rates during the COVID-19 pandemic did not find differences between the pandemic and the prepandemic eras or for the different COVID-19 periods; the combined consideration of live births and stillbirths did not change the degree of reduction in births (Table 3; eFigure 3 in Supplement 1).

**Table 1.** Changes in Birth Rates and Preterm Delivery Rates in 2017-2019 vs 2020

Period	Births, No. (%)			*P* value^a^
	2017-2019	2020	Total	
Total births				
Overall	140 346 (100)	44 481 (100)	184 827 (100)	.06
March 14 to May 15	28 975 (20.65)	9207 (20.7)	38 182 (20.66)	.81
May 16 to October 18	78 417 (55.87)	25 070 (56.36)	103 487 (55.99)	.07
October 19 to December 31	32 954 (23.48)	10 204 (22.94)	43 158 (23.35)	.02
Full term born infants (≥37 wk)				
Overall	128 262 (91.39)	40 943 (92.05)	169 205 (91.55)	.18
March 14 to May 15	26 457 (18.85)	8472 (19.05)	34 929 (18.9)	.78
May 16 to October 18	71 721 (51.1)	23 049 (51.82)	94 770 (51.27)	.18
October 19 to December 31	30 084 (21.44)	9422 (21.18)	39 506 (21.37)	.07
Late preterm infants (32-36 wk)				
Overall	9334 (6.65)	2751 (6.18)	12 085 (6.54)	.15
March 14 to May 15	1927 (1.37)	551 (1.24)	2478 (1.34)	.48
May 16 to October 18	5202 (3.71)	1589 (3.57)	6791 (3.67)	.06
October 19 to December 31	2205 (1.57)	611 (1.37)	2816 (1.52)	.12
Very preterm infants (<32 wk)				
Overall	2064 (1.47)	572 (1.29)	2636 (1.43)	.01
March 14 to May 15	445 (0.32)	144 (0.32)	589 (0.32)	.07
May 16 to October 18	1097 (0.78)	316 (0.71)	1413 (0.76)	.37
October 19 to December 31	522 (0.37)	112 (0.25)	634 (0.34)	.005

^a^ Calculated with Pearson test.

**Table 2.** Odds Ratios and Relative Risks for Very Preterm Birth at Less Than

Phase	Births, No.					OR (95% CI)		RR	*P* value^a^
	2020		2017-2019						
	<32 wk	≥37 wk	<32 wk	≥37 wk					
All	572	40 943	2064	128 262	0.87 (0.79-0.95)		0.87 (0.79-0.95)		.003
March 14 to May 15	144	8472	445	26 457	1.01 (0.83-1.22)		1.01 (0.84-1.21)		.92
May 16 to October 18	316	23 049	1097	71 721	0.90 (0.79-1.02)		0.90 (0.79-1.02)		.09
October 19 to December 31	112	9422	522	30 084	0.69 (0.55-0.84)		0.06 (0.56-0.84)		<.001

Abbreviations: OR, odds ratio; RR, relative risk.

^a^ Calculated with Fisher exact test.

**Table 3.** Maternal Baseline and Pregnancy Characteristics in 2017-2019 and 2020

Characteristic	Births, No. (%)			*P* value
	2017-2019	2020	Total	
**All births**				
No.	140 346	44 481	184 827	NA
Routine medical check-ups during pregnancy				
Median (IQR)	11 (10-13)	11 (9-13)	11 (10-13)	.15^a^
Missing, No.	35 496	10 931	46 427	NA
Maternal age, median (IQR), y	31 (28-35)	32 (28-35)	31 (28-35)	<.001^a^
GA at admission				
Median (IQR), wk	39 4/7 (38 3/7 to 40 2/7)	39 4/7 (38 4/7 to 40 3/7)	39 4/7 (38 3/7 to 40 2/7)	<.001^a^
Missing, No.	686	215	901	NA
Admission to birth interval, median (IQR), d	0 (0-1)	0 (0-1)	0 (0-1)	.28^a^
Antenatal steroids	6399 (4.56)	1481 (3.33)	7880 (4.26)	<.001^b^
Mode of delivery				
Spontaneous	93 711 (66.77)	29 736 (66.85)	123 447 (66.79)	.003^b^
Cesarean	46 635 (33.23)	14 745 (33.15)	61 380 (33.21)	.76^b^
Emergency cesarean delivery^c^	2071 (4.44)	708 (4.80)	2779 (4.53)	.07^b^
Stillbirth	548 (0.39)	171 (0.38)	719 (0.39)	.86^b^
**Full term infants (≥37 wk)**				
No.	128 262	40 943	169 205	NA
Routine medical check-ups during pregnancy				
Median (IQR)	11 (10-13)	11 (10-13)	11 (10-13)	.02^a^
Missing, No.	32 725	10 217	42 942	NA
Maternal age, median (IQR), y	31 (28-35)	32 (28-35)	31 (28-35)	<.001^a^
GA at admission, median (IQR), wk	39 5/7 (38 5/7 to 40 3/7)	39 5/7 (38 6/7-40 3/7)	39 5/7 (38 5/7 to 40 3/7)	.01^a^
Admission to birth interval, , median (IQR), d	0 (0-1)	0 (0-1)	0 (0-1)	.85^a^
Antenatal steroids	2024 (1.58)	330 (0.81)	2354 (1.39)	<.001^b^
Mode of delivery				
Spontaneous	88 419 (68.94)	28 164 (68.79)	116 583 (68.9)	<.001^b^
Cesarean	39 843 (31.06)	12 779 (31.21)	52 622 (31.10)	.57^b^
Emergency cesarean delivery^c^	1560 (3.92)	540 (4.23)	2100 (3.99)	.12^b^
Stillbirth	136 (0.11)	53 (0.13)	189 (0.11)	.22^b^
**Late preterm infants (32-36 wk)**				
No.	9334	2751	12 085	NA
Routine medical check-ups during pregnancy				
Median (IQR)	9 (8-11)	9 (8-11)	9 (8-11)	.49^a^
Missing, No.	1751	439	2190	NA
Maternal age, median (IQR), y	32 (29-36)	32 (29-36)	32 (29-36)	.65^a^
GA at admission, median (IQR), wk	35 4/7 (34 1/7 to 36 2/7)	35 4/7 (34 1/7-36 2/7)	35 3/7 (34 0/7-36 2/7)	.12^a^
Admission to birth interval, median (IQR), d	0 (0-2)	1 (0-2)	1 (0-2)	.44^a^
Antenatal steroids	2738 (29.33)	707 (25.70)	3445 (28.51)	<.001^b^
Mode of delivery				
Spontaneous	4272 (45.79)	1280 (46.53)	5554 (45.96)	.17^b^
Cesarean	5060 (54.21)	1471 (53.47)	6531 (54.04)	.49^b^
Emergency cesarean^c^	291 (5.75)	104 (7.07)	395 (6.05)	.06^b^
Stillbirth	137 (1.47)	41 (1.49)	178(1.47)	.93^b^
**Very preterm infants (<32 wk)**				
No	2064	572	2636	NA
Routine medical check-ups during pregnancy				
Median (IQR), No.	6 (5-8)	6 (5-8)	6 (5-8)	.90^a^
Missing, No.	44	96	544	NA
Maternal age, median (IQR), y	32 (28-36)	32 (29-36)	32 (28-36)	.65^a^
GA at admission, median (IQR), wk	27 2/7 (24 3/7 to 29 6/7)	27 2/7 (24 5/7 to 29 5/7)	27 2/7 (24 4/7 to 29 6/7)	.54^a^
Admission to birth interval, d	3 (1-9)	2 (1-8)	3 (1-9)	.18^a^
Antenatal steroids	1624 (78.67)	441 (77.10)	2065 (78.34)	.42^b^
Mode of delivery				
Spontaneous	550 (26.65)	147 (25.70)	697 (26.44)	.04^b^
Cesarean	1514 (73.35)	425 (74.30)	1939 (73.56)	.65^b^
Emergency cesarean delivery^c^	206 (13.60)	61 (14.35)	267 (13.77)	.69^b^
Stillbirth	267 (12.94)	76 (13.29)	343 (13.01)	.83^b^

Abbreviations: GA, gestational age; NA, not applicable.

^a^ Test used: Wilcoxon test.

^b^ Test used: Pearson test.

^c^ Relative frequency is calculated in relation to all cesarean deliveries.

### Maternal and Neonatal Characteristics Before and During the COVID-19 Pandemic

There were no clinically relevant differences between the pandemic and prepandemic eras for the total cohort or among preterm births in prenatal care characteristics, including the number of routine medical check-ups during pregnancy, gestational age at admission, period between admission to hospital and delivery, ANS application, mode of delivery, and frequency of emergency cesarean delivery (Table 3). Among neonatal characteristics, there were no differences in gestational age, birth weight and head circumference at birth, sex, or singleton vs multiple birth between the COVID-19 pandemic era and the corresponding periods in 2017 to 2019. Furthermore, blood gas parameters of pH and negative base excess and Apgar scores at 1, 5, and 10 minutes after birth did not differ. Separate consideration of the preterm infant population rendered the same results (Table 4).

**Table 4.** Neonatal Characteristics in 2017-2019 and 2020

Characteristic	2017-2019	2020	Total	*P* value
**All births**					
No.	140 346	44 481	184 827	NA
Gestational age at birth				
Median (IQR), wk	39 4/7 (38 4/7 to 40 3/7)	39 4/7 (38 4/7 to 40 3/7)	39 4/7 (38 4/7 to 40 3/7)	<.001^a^
Missing, No.	686	215	901	NA
Birth weight, g	3380 (3040 to 3700)	3400 (3065 to 3710)	3380 (3050 to 3700)	<.001^a^
Head circumference at birth				
Median (IQR), cm	35.00 (34.00 to 36.00)	35.00 (34.00 to 36.00)	35.00 (34.00 to 36.00)	.52^a^
Missing, No.	51 880	24 249	76 129)	NA
Sex, No. (%)				
Male	72 215 (51.45)	22 831 (51.33)	95 046 (51.42)	.09^c^
Female	68 109 (48.53)	21 641 (48.65)	89 750 (48.56)	
Diverse^b^	0	2 (<0.01)	2 (<0.01)	
Undefined	22 (0.02)	7 (0.02)	29 (0.02)	
Multiples, No. (%)	5451 (3.88)	1662 (3.74)	7113 (3.85)	.16^c^
Umbilical cord blood gas				
pH				
Median (IQR),	7.28 (7.22 to 7.34)	7.28 (7.21 to 7.33)	7.28 (7.22 to 7.33)	<.001^a^
Missing, No.	1485	483	1986	NA
Base excess				
Median (IQR)	−3.70 (−6.30 to −1.40)	−3.80 (−6.40 to −1.50)	−3.70 (−6.30 to −1.50)	<.001^a^
Missing	1834	484	2318	NA
Apgar score				
1 Min				
Median (IQR)	9 (9 to 9)	9 (9 to 9)	9 (9 to 9)	.19^a^
Missing	563	185	748	NA
5 Min				
Median (IQR)	10 (10 to 10)	10 (10 to 10)	10 (10 to 10)	<.001^a^
Missing	627	193	820	NA
10 Min				
Median (IQR)	10 (10 to 10)	10 (10 to 10)	10 (10 to 10)	.02^a^
Missing	733	216	949	NA
**Full term infants (≥37 wk)**					
No.	128 262	40 943	169 205	
Gestational age at birth, median (IQR), wk	39 5/7 (38 6/7 to 40 4/7)	39 5/7 (38 6/7 to 40 4/7)	39 5/7 (38 6/7 to 40 4/7)	.02^a^
Birth weight, median (IQR), g	3420 (3130 to 3725)	3440 (3145 to 3740)	3425 (3130 to 3730)	<.001^a^
Head circumference at birth				
Median (IQR), cm	35.00 (34.00 to 36.00)	35.00 (34.00 to 36.00)	35.00 (34.00 to 36.00)	.89^a^
Missing, No.	46 125	22 037	68 162	NA
Sex, No. (%)				
Male	65 562 (51.12)	20 893 (51.03)	86 455 (51.09)	.06^c^
Female	62 682 (48.87)	20 045 (48.96)	82 727 (48.89)	
Diverse^b^	0	2 (<0.01)	2 (<0.01)	
Undefined	18 (0.01)	3 (0.01)	21 (0.01)	
Multiples, No. (%)	2457 (1.92)	748 (1.83)	3205 (1.89)	.25^c^
Umbilical cord blood gas				
pH				
Median (IQR)	7.28 (7.22 to 7.33)	7.27 (7.21 to 7.33)	7.28 (7.22 to 7.33)	<.001^a^
Missing, No.	815	291	1106	NA
Base excess				
Median (IQR)	−3.80 (−6.40 to −1.50)	−3.90 (−6.50 to −1.60)	−3.80 (−6.40 to −1.50)	<.001^a^
Missing, No.	1132	292	1424	NA
Apgar score				
1 Minute				
Median (IQR)	9 (9 to 9)	9 (9 to 9)	9 (9 to 9)	.13^a^
Missing, No.	223	75	298	NA
5 Minutes				
Median (IQR)	10 (10 to 10)	10 (10 to 10)	10 (10 to 10)	<.001^a^
Missing, No.	234	69	303	NA
10 Minutes				
Median (IQR)	10 (10 to 10)	10 (10 to 10)	10 (10 to 10)	<.001^a^
Missing, No.	280	84	364	NA
**Late preterm infants (32 to 36 wk)**					
No.	9334	2751	12 085	
Gestational age at birth, median (IQR), wk	35 5/7 (34 4/7 to 36 3/7)	35 5/7 (34 4/7 to 36 3/7)	35 5/7 (34 4/7 to 36 3/7)	.45^a^
Birth weight, median (IQR), g	2500 (2150 to 2830)	2490 (2142 to 2800)	2500 (2150 to 2820)	.32^a^
Head circumference at birth				
Median (IQR), cm	33.00 (32.00 to 34.00)	33.00 (32.00 to 34.00)	33.00 (32.00 to 34.00)	.68^a^
Missing, No.	4055	1606	5661	NA
Sex, No. (%)				
Male	5175 (55.44)	1534 (55.76)	6709 (55.52)	.13^c^
Female	4157 (44.54)	1214 (44.13)	5371 (44.54)	
Undefined	2 (0.02)	3 (0.11)	5 (0.04)	
Multiples, No. (%)	2333 (24.99)	757 (27.52)	3090 (25.57)	.008^c^
Umbilical cord blood gas				
pH				
Median (IQR)	7.30 (7.24 to 7.35)	7.30 (7.24 to 7.34)	7.30 (7.24 to 7.34)	.006^a^
Missing, No.	232	72	304	NA
Base excess				
Median (IQR)	−2.90 (−5.40 to −0.90)	−3.00 (−5.50 to −1.00)	−2.90 (−5.40 to −1.00)	.18^a^
Missing, No.	262	72	334	NA
Apgar score				
1 Minute				
Median (IQR)	9 (8 to 9)	9 (8 to 9)	9 (8 to 9)	.005^a^
Missing, No.	118	44	162	NA
5 Minutes				
Median (IQR)	10 (9 to 10)	10 (9 to 10)	10 (9 to 10)	.003^a^
Missing, No.	143	51	194	NA
10 Minutes				
Median (IQR)	10 (10 to 10)	10 (9 to 10)	10 (9 to 10)	.16^a^
Missing, No.	261	54	216	NA
**Very preterm infants (<32 wk)**					
No.	2064	572	2636	NA
Gestational age at birth, median (IQR), wk	28 5/7 (26 1/7 to 30 4/7)	28 5/7 (25 6/7 to 30 4/7)	28 5/7 (26 0/7 to 30 4/7)	.80^a^
Birth weight, median (IQR), g	1090 (740 to 1440)	1091 (739 to 1446)	1090 (740 to 1440)	.83^a^
Head circumference at birth, cm				
Median (IQR), cm	26.50 (23.50 to 28.15)	26.00 (23.00 to 29.00)	26.30 (23.00 to 28.50)	.49^a^
Missing, No.	1257	410	1667	NA
Sex, No. (%)				
Male	1132 (54.84)	292 (51.05)	1424 (54.02)	.25^c^
Female	930 (45.06)	279 (48.78)	1209 (45.86)	
Undefined	2 (0.10)	1 (0.17)	3 (0.11)	
Multiples, No. (%)	646 (31.3)	157 (27.4)	803 (30.5)	.08^c^
Umbilical cord blood gas				
pH				
Median (IQR)	7.32 (7.26 to 7.36)	7.31 (7.25 to 7.36)	7.32 (7.26 to 7.36)	.05^a^
Missing, No.	418	119	537	NA
Base excess				
Median (IQR)	−2.70 (−5.10 to −0.80)	−2.60 (−4.70 to −0.60)	−2.70 (−5.10 to −0.80)	.28^a^
Missing, No.	419	119	538	NA
Apgar score				
1 Minute				
Median (IQR)	7 (5 to 8)	7 (4 to 8)	7 (5 to 8)	.07^a^
Missing, No.	216	65	361	NA
5 Minutes				
Median (IQR)	8 (7 to 9)	8 (6 to 9)	8 (7 to 9)	.25^a^
Missing, No.	243	72	315	NA
10 Minutes				
Median (IQR)	9 (8 to 9)	9 (8 to 9)	9 (8 to 9)	.05^a^
Missing, No.	284	77	361	NA

Abbreviation: NA, not applicable.

^a^ Tests used: Wilcoxon test.

^b^ Diverse is a legal option for sex assignment at birth in Germany for intersex infants when assignment to either male or female is not completely clear.

^c^ Tests used: Pearson test.

### Maternal Risk Factors for Preterm Delivery

When comparing maternal baseline characteristics for the total cohort before and during the COVID-19 pandemic and applying adjustment for multiple testing, no differences were detected for maternal age older than 35 years, history of preterm birth, history of previous cesarean delivery or uterine surgery, or complications during previous pregnancies. However, the odds of VPT birth were decreased in pregnancies with a history of maternal serious disease (OR, 0.64; 95% CI, 0.50-0.83), while other factors, such as a family history of serious disease, diabetes during pregnancy, maternal obesity, serious or severe psychological burden during pregnancy, preexisting hypertension, bleeding at less than 28 weeks or at 28 weeks or later, placenta previa, and multiple pregnancies, remained unchanged (Figure, A; eTable 3 in Supplement 1).^13^ Analyses of the total cohort of preterm infants born at less than 37 weeks found the same results, aside from maternal age older than 35 years. When only considering preterm infants born at 32 0/7 to 36 6/7 weeks, maternal age older than 35 years remained a risk factor but not a history of maternal serious disease (eTable 4 and eTable 5 in Supplement 1).

**Figure.**
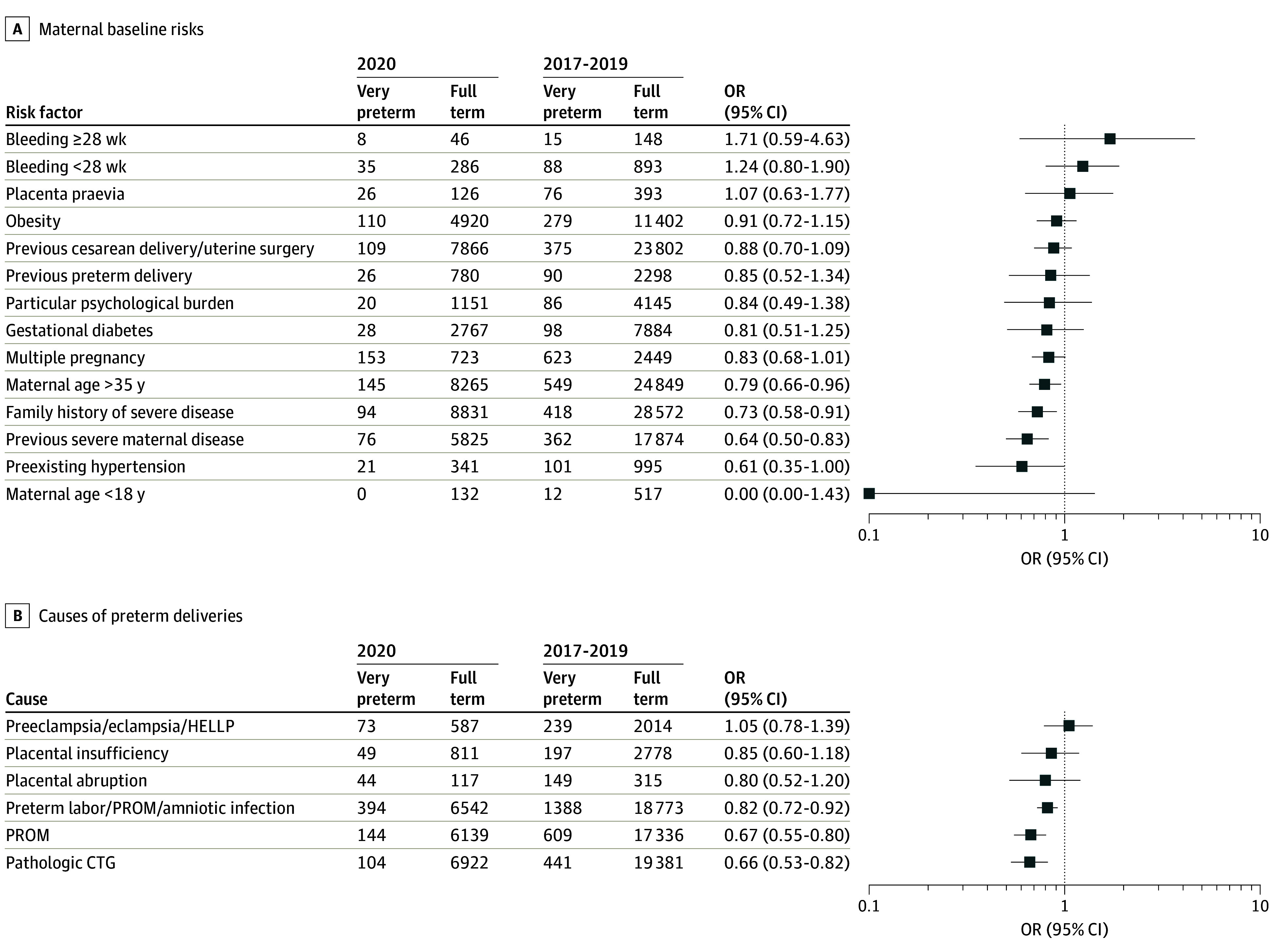
Changes of Maternal Baseline Risks and Causes of Preterm Deliveries During the COVID-19 Pandemic Analyses of maternal baseline risks (A) and causes of preterm deliveries (B) from the quality-assurance registry of Hesse, Germany, comparing the prepandemic period (2017-2019) and the COVID-19 pandemic (2020) in preterm births at less than 32 weeks are presented as odds ratios (ORs) and 95% CIs. CTG indicates cardiotocography; HELLP, hemolysis, elevated liver enzymes, low platelet count; PROM, premature rupture of membranes

### Causes of Preterm Births Before and During the COVID-19 Pandemic

Lastly, we evaluated the variations in causes of preterm birth before and during the pandemic. While preterm birth for maternal hypertension; preeclampsia, eclampsia, or HELLP syndrome; placental insufficiency with intrauterine growth restriction; and placental disruption did not change, the frequency of births for intrauterine infection was reduced among VPT infants during the pandemic. Furthermore, odds of births for pathologic CTG were lower (OR, 0.66; 95% CI, 0.53-0.82) (Figure, B; eTable 3 in Supplement 1). The reductions of preterm deliveries at less than 32 weeks were comparable in the categories of all indicated and nonindicated births (eFigure 4 in Supplement 1). Lastly, we studied the associations of maternal age, pathological CTG, and indicated birth with our outcomes of interest (eTable 6 in Supplement 1). Again, the evaluation of all preterm infants born at less than 37 weeks and of those delivered at 32 0/7 weeks to 36 6/7 weeks rendered congruent results for intrauterine infection, while pathologic CTG was not a significant cause of birth in this patient population (eTable 4 and eTable 5 in Supplement 1).

## Discussion

This population-based, descriptive, epidemiological cohort study of more than 180 000 births from the federal state of Hesse, Germany, had 2 key findings that are of importance for future studies on the variations in VPT birth rates during the COVID-19 pandemic and for all future research efforts intended to reduce the rate of preterm births. First, we mirrored the findings from previous studies that strict lockdown measures were associated with a reduction of preterm birth rates, but we found changing dynamics of this association and increased associations with later stages of the pandemic, including restrictions of normal work and private life.^12^ This finding is relevant, as the longer duration of altered exposure might have implications for future research strategies to prevent preterm birth; furthermore, this finding argues for taking into consideration the total duration of pregnancy and not solely the third trimester. This consideration has not been fully examined by other studies that compared premature birth rates during the COVID-19 pandemic period with the prepandemic period. Additionally, we did not observe the previously reported shift toward higher gestational ages within the population of preterm infants.^26^ Second, we observed relevant changes in maternal risks and causes for preterm birth, including lower rates of history of own serious disease and delivery for intrauterine infection, while other factors, like bleeding during pregnancy, multiple pregnancy, psychological distress, and preeclampsia, eclampsia, or HELLP syndrome, were not altered. This was reflected by the comparable reduction in indicated and nonindicated preterm deliveries. We postulate that measures enacted during the pandemic to mitigate the spread of COVID-19 had differing levels of impact for the different risk factors and causes of preterm birth. Our registry-based analyses do not allow us to assess variations in clinicians’ attitudes toward delivery in nonemergency situations, but maternal and fetal emergency situations should not have changed the indication for delivery. Overall, these results demand separate consideration of the different risks and causes of preterm birth.

### Data Interpretation in the Context of the Hygiene Hypothesis

Our finding of decreased incidence of preterm births for intrauterine infection might indicate that the contact and hygiene restrictions during the COVID-19 pandemic also reduced preterm births. Alternatively, the very strict lockdown measures and stay-at-home orders might have reduced individual stress levels during pregnancy, particularly with respect to work and leisure activity stressors, and the association of this potentially reduced stress with preterm birth has not been evaluated thoroughly.^27,28^ As the number of routine medical check-ups during pregnancy in the ambulatory sector was unchanged, our results argue against lower utilization of preventive health care services. The unchanged time intervals from admission to birth, the constant rate of ANS application, and the constant distribution of modes of delivery argue against altered preclinical medical care use, delayed hospital admissions, or changes in hospital care practices as observed in other disease entities and emergency situations during the COVID-19 pandemic. This might be due to individuals’ own estimation of their vulnerability related to pregnancy, COVID-19, and other health factors, which did not lead to a change in behaviors for seeking medical care.^29,30,31,32^ It is encouraging that the direction of health care resources toward the treatment of patients with COVID-19 infection did not result in neglect or lower prioritization of pregnant individuals admitted for immanent preterm birth by hospital staff. In these points, our results are broadly in line with other publications on this topic.^10,33,34^

### Measurements for Preterm Birth Prevention

While improvements in postnatal preterm care have dramatically improved survival and outcomes for preterm infants during the last decades, efforts to reduce the rate of preterm deliveries have not resulted in comparable improvements, and rates of preterm births continue to increase.^35,36,37^ While baseline risk factors, including maternal age older than 35 years, multiple pregnancies, and the use of medical reproductive measures, particularly assisted reproductive technologies, are nonmodifiable except by increasing awareness and education on the heightened risks, secondary obstetric preventive measures so far have failed to reduce the risk of preterm births. Particularly disappointingly, all the secondary prevention measures and strategies to prolong the duration of pregnancy in patient populations at risk of preterm birth, including cervical pessary or cerclage, vaginal progesterone, and the use of tocolytic agents, have had low efficacy in reducing rates of preterm birth.^38,39,40^ Therefore, this obstetric challenge remains unresolved. Our findings of a reduction in the overall rate of preterm birth at less than 37 weeks’ gestation for high-risk pregnancies, including maternal age older than 35 years and history of serious maternal disease, during the COVID-19 pandemic might be indicative of a reduced preparedness of women with baseline risk factors to get pregnant or lower frequency of pregnancies following artificial fertilization. This suggests that primary preventative measures could be effective in reducing the risk of premature birth. While our datasets do not allow to specify which lockdown measures in the working environment and private life may account for this reduction, our results justify further elaboration within future prospective studies. Primary preventive measures might be more effective than prolonging the gestation in pregnancies with evident risks. It remains speculative how the drop in pathologic CTG rates during the COVID-19 pandemic can be explained. An Australian analysis provides a potential explanation: there was a decrease in iatrogenic preterm deliveries for fetal compromise, including pathologic CTG cases, and a simultaneous increase in stillbirths, and we cannot exclude underdocumentation of such events during the COVID-19 pandemic. One further explanation might be that pathologic CTG is a part of a larger risk constellation and thereby might reflect pregnancies in which no underlying baseline risks were documented. Our divergent results for the different risk categories and causes for preterm births indicate that there will be no one-size-fits-all strategy. Different approaches and focus on prespecified relevant clinical outcomes will be necessary, as is already established other clinical practices, such as in oncologic therapies.^41,42^ The COVID-19 lockdown measures were not associated with reduced rates of prematurity for other baseline risk constellations, such as preeclampsia, eclampsia, and HELLP syndrome, as reported elsewhere.^13^ These risks are not predetermined by modifiable baseline characteristics that may have been impacted by the pandemic, suggesting more work is needed to find efficient screening and prevention measures that can be applied in the general public.

### Strengths and Limitations

Our descriptive epidemiological cohort study has several important strengths, including the use of a population-level study cohort from a region that is broadly representative of routine medical care during pregnancy and the risks and preventive measures for preterm birth in Germany and Europe. Due to the obligatory documentation within the quality assurance registry, the database has complete coverage, and the sample size of preterm births is larger than in most previous reports on this topic.^2,7,8,9^ Furthermore, a broad range of clinically relevant and patient-focused measures were available that were collected based on prespecified and pretested items. Additionally, we were able to exclude carry-over effects by changes in the stillbirth rate.

This study has several limitations. First, while the patient data collection was complete, we cannot exclude underreporting of pregnancy risks, as these items were not individually queried but were collected within 1 section of the documentation system. We also cannot exclude individuals who had more than 1 preterm delivery during the COVID-19 pandemic, but this should have been a rare event. Second, the introduction of a national guideline on the prevention and management of preterm birth in 2019 may have affected outcomes of this study, but it is unlikely that the categories of causes of preterm deliveries with declines were influenced to such an extent, and the implementation of these new guidelines may have been delayed due to the pandemic. Most importantly, the rate of preterm births at less than 32 weeks returned to the prepandemic level after the official end of the pandemic in Germany. Third, changes in psychological states are well described for the COVID-19 pandemic. In our analysis, we did not see an increased psychological stress burden, which might be due to underreporting from patients themselves.^43^ Fourth, we cannot exclude residual confounding by undocumented characteristics, such as physical activity, maternal diet, nicotine or alcohol consumption during pregnancy, maternal body mass index, and availability of prevention programs of preterm delivery before and during the pandemic. Fifth, we were not able to assess variations in societal stressors during pregnancy or in SARS-CoV-2 infection rates during pregnancy. Sixth, the sample did not allow us to examine outcomes by monthly intervals to describe changes over time. Furthermore, we cannot assess whether and how increasing concern regarding the COVID-19 pandemic impacted the behavior of pregnant individuals before the lockdown measures were enacted. Therefore, we decided not to introduce further subcategories. Additionally, we studied pregnancies and preterm births from 1 region, so our findings should be assessed against other independent cohorts during the COVID-19 pandemic. Undocumented changes in lifestyle, exposure to infectious agents besides COVID-19, and societal behavior change associated with the COVID-19 pandemic might also have contributed to the results.

## Conclusions

The findings of this cohort study suggest that there was no deterioration of medical care during pregnancy or immediately before preterm delivery, which allowed us to observe the reduction in preterm birth rates during the COVID-19 pandemic. Importantly, the duration of exposure to the strict lockdown measures and stay-at-home orders was associated with the degree of reduction in preterm births. The combined analyses of all available datasets revealed that primary care measures were associated with reduced risk for preterm birth in several categories of high-risk pregnancies. The unchanged level of psychological burden documented during pregnancy suggests that it may be worthwhile to study measures to reduce maternal stress in efforts to decrease risk of preterm births for intrauterine infection and that research should focus on reducing rates of preterm deliveries in pregnancies among individuals with a history of serious disease. Our findings add novel information on the epidemiology of the COVID-19 pandemic in association with preterm birth prevention and suggest that further research efforts on primary preventive measures and different causes of preterm delivery are justified and promising.

## Notes

Supplement 1. **eFigure 1.** Diagram of the Pandemic Phases and Corresponding Prepandemic Time Periods **eFigure 2.** Flowchart of the Total Sample and Patient Subpopulations Included in the Study **eFigure 3.** Livebirths, Preterm Births <32 Weeks’ Gestation, and Stillbirths by Year (2017-2020) **eFigure 4.** Indicated and Nonindicated Preterm Births (<32 Weeks) Before and During the COVID-19 Pandemic **eTable 1.** Time Effect With and Without the Year 2020 (COVID-19 Lockdown) **eTable 2.** Odds Ratios for Prematurity (<37 Weeks) in Different Phases of the Year **eTable 3.** Pregnancy and Birth Risks in 2017-2019 and 2020 in Very Preterm (<32 Weeks) Infants **eTable 4.** Pregnancy and Birth Risks in 2017-2019 and 2020 in Preterm (<37 Weeks) Infants **eTable 5.** Pregnancy and Birth Risks in 2017-2019 and 2020 in Late Preterm (32 to 36 Weeks) Infants **eTable 6.** Logistic Regression Models for Very Preterm Births <32 Weeks vs Term-Born Infants With Covariates

Supplement 2.
Data Sharing Statement
