# Supplementary material for: Characteristics and Rates of Preterm Births During the COVID-19 Pandemic in Germany
Source: JAMA Netw Open. 2024 Sep 10;7(9):e2432438. doi: 10.1001/jamanetworkopen.2024.32438 (PMC11388025; doi:10.1001/jamanetworkopen.2024.32438)
Supplement: Supplement 1. — eFigure 1. Diagram of the Pandemic Phases and Corresponding Prepandemic Time Periods eFigure 2. Flowchart of the Total Sample and Patient Subpopulations Included in the Study eFigure 3. Livebirths, Preterm Births <32 Weeks’ Gestation, and Stillbirths by Year (2017-2020) eFigure 4. Indicated and Nonindicated Preterm Births (<32 Weeks) Before and During the COVID-19 Pandemic eTable 1. Time Effect With and Without the Year 2020 (COVID-19 Lockdown) eTable 2. Odds Ratios for Prematurity (<37 Weeks) in Different Phases of the Year eTable 3. Pregnancy and Birth Risks in 2017-2019 and 2020 in Very Preterm (<32 Weeks) Infants eTable 4. Pregnancy and Birth Risks in 2017-2019 and 2020 in Preterm (<37 Weeks) Infants eTable 5. Pregnancy and Birth Risks in 2017-2019 and 2020 in Late Preterm (32 to 36 Weeks) Infants eTable 6. Logistic Regression Models for Very Preterm Births <32 Weeks vs Term-Born Infants With Covariates [file jamanetwopen-e2432438-s001.pdf]

Supplemental Online Content

Staude B, Misselwitz B, Louwen F, et al. Characteristics and rates of preterm births during the COVID-19 pandemic in Germany. *JAMA Netw Open*. 2024;7(9):e2432438. doi:10.1001/jamanetworkopen.2024.32438

eFigure 1. Diagram of the Pandemic Phases and Corresponding Prepandemic Time Periods
eFigure 2. Flowchart of the Total Sample and Patient Subpopulations Included in the Study
eFigure 3. Livebirths, Preterm Births <32 Weeks' Gestation, and Stillbirths by Year (2017-2020)
eFigure 4. Indicated and Nonindicated Preterm Births (<32 Weeks) Before and During the COVID-19 Pandemic
eTable 1. Time Effect With and Without the Year 2020 (COVID-19 Lockdown)
eTable 2. Odds Ratios for Prematurity (<37 Weeks) in Different Phases of the Year
eTable 3. Pregnancy and Birth Risks in 2017-2019 and 2020 in Very Preterm (<32 Weeks) Infants
eTable 4. Pregnancy and Birth Risks in 2017-2019 and 2020 in Preterm (<37 Weeks) Infants
eTable 5. Pregnancy and Birth Risks in 2017-2019 and 2020 in Late Preterm (32 to 36 Weeks) Infants
eTable 6. Logistic Regression Models for Very Preterm Births <32 Weeks vs Term-Born Infants With Covariates

This supplemental material has been provided by the authors to give readers additional information about their work.

© 2024 Staude B et al. *JAMA Network Open.*

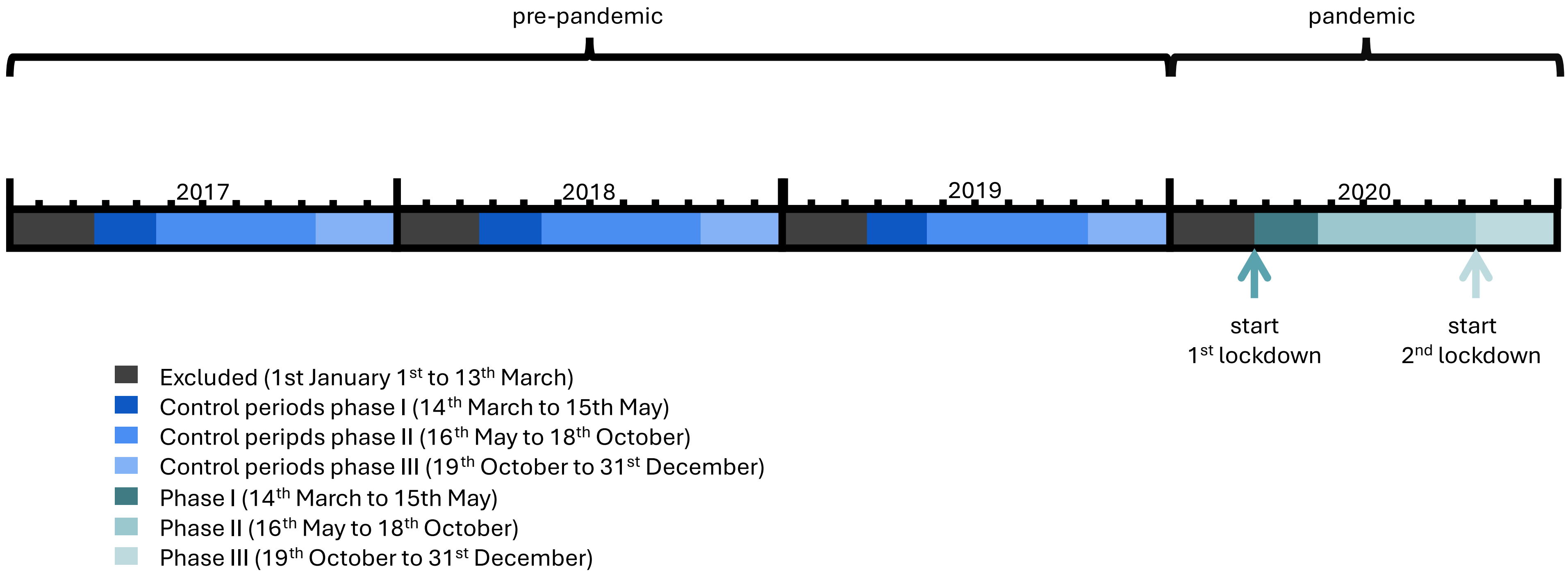

eFigure1

© 2024 Staude B et al. *JAMA Network Open.*

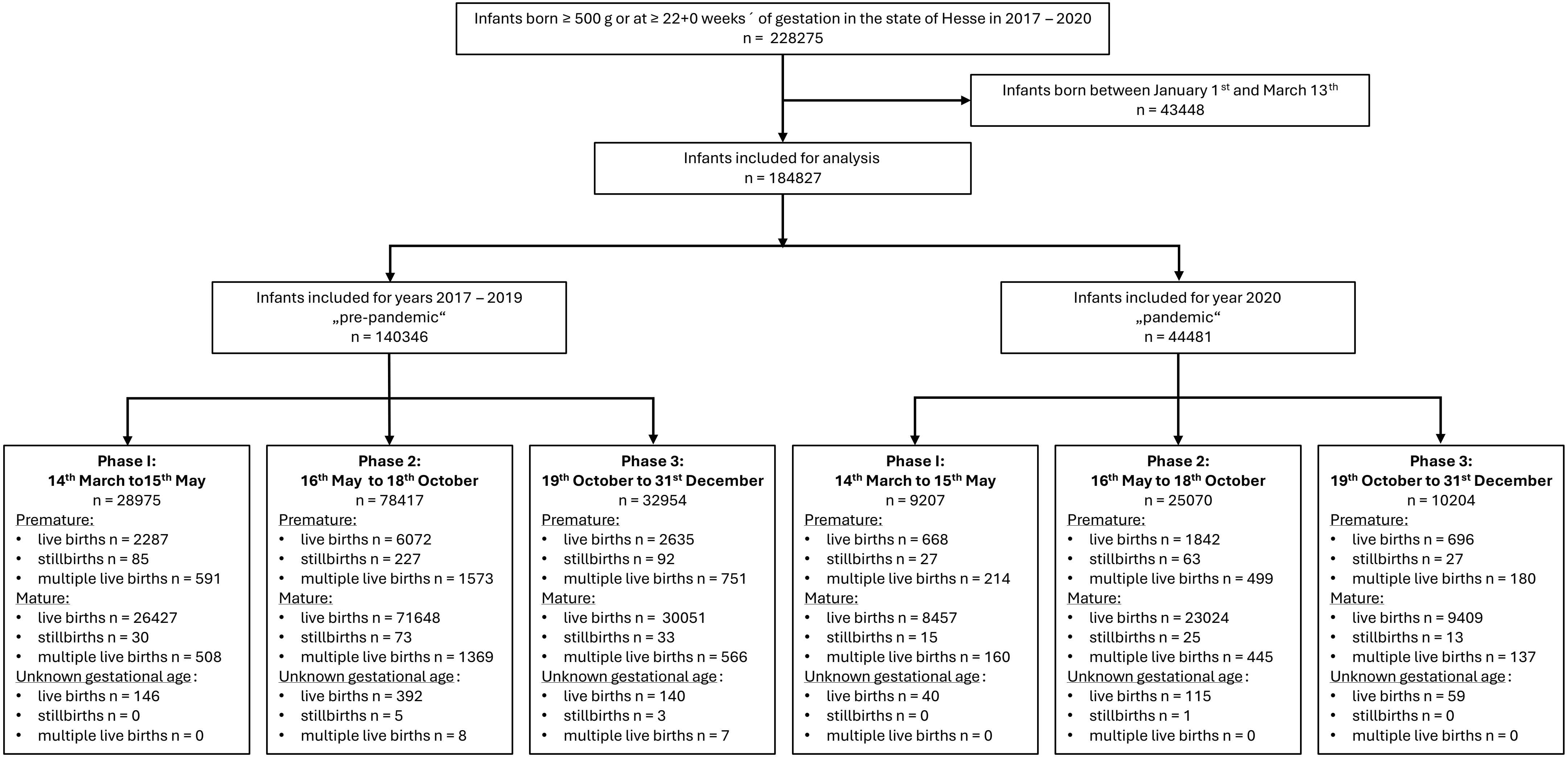

eFigure2

© 2024 Staude B et al. *JAMA Network Open.*

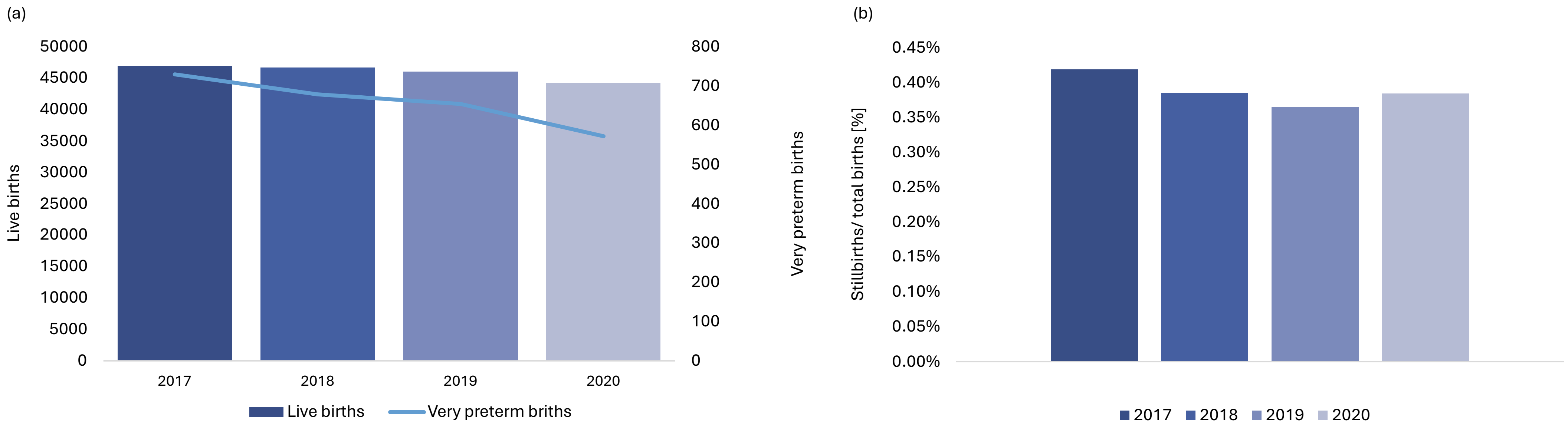

eFigure 3

© 2024 Staude B et al. *JAMA Network Open.*

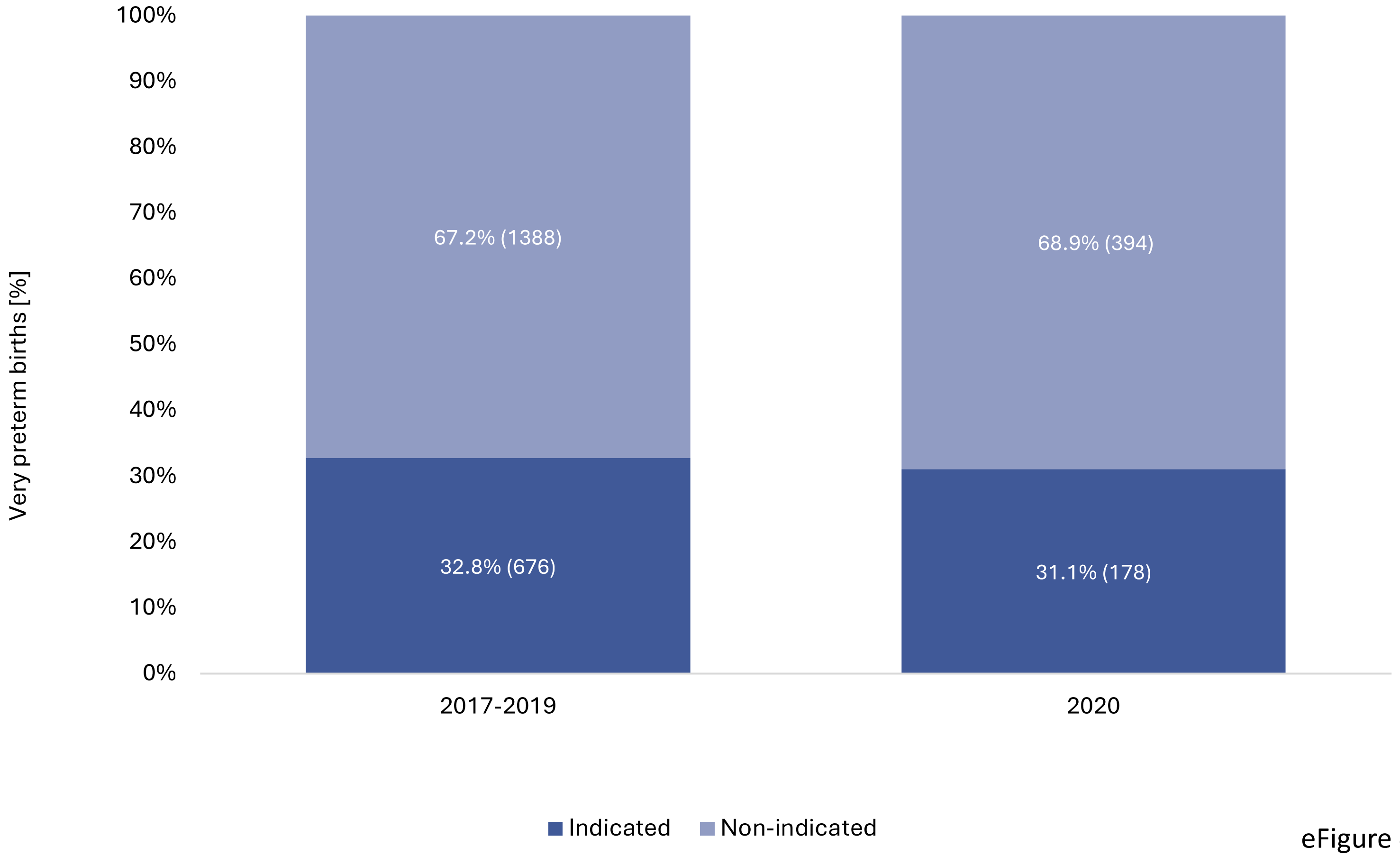

eFigure 4

eFigure 4

© 2024 Staude B et al. *JAMA Network Open.*

eFigure Legends

eFigure 1. Diagram of the pandemic phases and corresponding pre-pandemic time periods. Depicted are the different phases of the first (phase I; 14/03/2020 to 15/05/2020) and second (phase III; 19/10/2020 to 31/12/2020) lockdown and the less vigorous restrictions in between (phase II; 16/05/2020 to 18/10/2020) and the corresponding pre-pandemic periods separated by years.

eFigure 2. Flow chart of the total sample and patient subpopulations included into the analyses of the study. Presentation of case numbers included into the analyses separated into the pre-pandemic (2017-2020) and pandemic (2020) period. Numbers are segregated for term and preterm livebirths, stillbirths and multiple live births and for cases with unknown gestational age.

eFigure 3. Livebirths, preterm births <32 weeks´ gestation and stillbirths by year (2017-2020). In (a), numbers of total live births and preterm deliveries <32 weeks are given by year. In (b), the yearly frequency of stillbirths is presented.

eFigure 4. Indicated and non-indicated preterm births <32 weeks before and during the COVID-19 pandemic. Proportions of infants with non-indicated deliveries with spontaneous onset by preterm labor, premature rupture of membranes (PROM) or amniotic infection and indicated births comprising all other causes of preterm delivery are given for the pre-pandemic (2017-2019) situation and the COVID-19 pandemic (2020) are given.

© 2024 Staude B et al. *JAMA Network Open.*

Supplemental Tables

eTable 1: Time effect with and without the year 2020 (COVID-19 lockdown)

	**Pre-pandemic**			**Pandemic**	
Predictors	*Odds Ratios*	*(CI)*	*p*	*Odds Ratios(CI)*	*p*
(Intercept)	0.02 (0.02 – 0.02)		**<0.001**	0.02 (0.02 – 0.02)	**<0.001**
Year of birth	0.96 (0.91 – 1.01)		0.100	0.94 (0.91 – 0.98)	**0.001**
Observations	130326			171841	
AIC	21208.6			27246.8	

Logistic regression models were calculated for the occurrence of very preterm deliveries vs term deliveries in the years before the COVID lockdown (2017-2019) and including the year 2020. CI, confidence interval; AIC, Akaike Information Criterion.

© 2024 Staude B et al. *JAMA Network Open.*

eTable 2. Odds ratios for prematurity (< 37 weeks) in different phases of the year

	2020		2017 - 2019		odds ratio			Fisher exact test
Phase	preterm	term	preterm	term	OR	lower CI	upper CI	p-value
all	3323	40943	11398	128262	0.913	0.877	0.951	< 0.001
I	695	8472	2372	26457	0.915	0.837	1	0.05
II	1905	23049	6299	71721	0.941	0.892	0.993	0.03
III	723	9422	2727	30084	0.847	0.776	0.922	< 0.001

OR: odds ratio, CI: confidence interval; p-value from Fisher's Exact test

© 2024 Staude B et al. *JAMA Network Open.*

eTable 3. Pregnancy and birth risks in 2017-2019 and 2020 in very preterm infants (< 32 weeks)

	**2020**		**2017 - 2019**		**odds ratio**			**Fisher exact test**	
	very preterm	term	very preterm	term	OR	lower CI	upper CI	p-value	p-adjust
Multiple pregnancy	153	723	623	2449	0.83	0.68	1.01	0.07	1
Maternal age									
< 18 years	0	132	12	517	0	0	1.43	0.14	1
> 35 years	145	8265	549	24849	0.80	0.66	0.96	0.02	0.44
Previous severe maternal disease	76	5825	362	17874	0.64	0.50	0.83	<0.001	0.01
Previous caesarian section/uterine surgery	109	7866	375	23802	0.88	0.70	1.09	0.27	1
Previous preterm delivery	26	780	90	2298	0.85	0.52	1.34	0.52	1
Family history of severe disease	94	8831	418	28572	0.73	0.58	0.91	0.005	0.17
Gestational diabetes	28	2767	98	7884	0.81	0.51	1.25	0.36	1
Obesity	110	4920	279	11402	0.91	0.72	1.15	0.47	1
Particular psychological burden	20	1151	86	4145	0.84	0.49	1.38	0.55	1
Preexisting hypertension	21	341	101	995	0.61	0.35	1.00	0.05	1
Preeclampsia/eclampsia/HELLP	73	587	239	2014	1.05	0.78	1.39	0.72	1
Bleeding < 28 weeks	35	286	88	893	1.24	0.80	1.90	0.32	1
Bleeding ≥ 28 weeks	8	46	15	148	1.71	0.59	4.63	0.31	1
Placenta praevia	26	126	76	393	1.07	0.63	1.77	0.80	1
Placental abruption	44	117	149	315	0.80	0.52	1.20	0.28	1
Placental insufficiency	49	811	197	2778	0.85	0.60	1.18	0.39	1
Preterm labor/PROM/amniotic infection	394	6542	1388	18773	0.82	0.72	0.92	<0.001	0.01
PROM	144	6139	609	17336	0.67	0.55	0.80	<0.001	<0.001
Pathologic CTG	104	6922	441	19381	0.66	0.53	0.82	<0.001	0.003

OR: odds ratio, CI: confidence interval; p-value from Fisher's Exact test, p-value adjustment: Holm.
PROM: premature rupture of membranes. CTG: cardiotocography.

© 2024 Staude B et al. *JAMA Network Open.*

eTable 4. Pregnancy and birth risks in 2017-2019 and 2020 in preterm infants (< 37 weeks)

	**2020**		**2017 - 2019**		**odds ratio**			**Fisher exact test**	
	preterm	term	preterm	term	OR	lower CI	upper CI	p-value	p-adjust
Multiple pregnancy	884	723	2930	2449	1.02	0.91	1.15	0.71	1
Maternal age									
< 18 years	7	132	51	517	0.54	0.20	1.23	0.17	1
> 35 years	797	8265	2861	24849	0.84	0.77	0.91	<0.001	0.001
Previous severe maternal disease	575	5825	2063	17874	0.86	0.78	0.94	0.001	0.04
Previous caesarian section/uterine surgery	685	7866	2385	23802	0.87	0.79	0.95	0.002	0.05
Previous preterm delivery	179	780	538	2298	0.98	0.81	1.19	0.85	1
Family history of severe disease	724	8831	2652	28572	0.88	0.81	0.96	0.004	0.12
Gestational diabetes	249	2767	888	7884	0.80	0.69	0.93	0.003	0.07
Obesity	512	4920	1295	11402	0.92	0.82	1.02	0.12	1
Particular psychological burden	97	1151	471	4145	0.74	0.58	0.93	0.01	0.22
Preexisting hypertension	87	341	366	995	0.69	0.53	0.91	0.006	0.16
Preeclampsia/eclampsia/HELLP	295	587	1070	2014	0.95	0.80	1.11	0.52	1
Bleeding < 28 weeks	77	286	215	893	1.12	0.82	1.51	0.45	1
Bleeding ≥ 28 weeks	32	46	94	148	1.10	0.63	1.90	0.79	1
Placenta praevia	104	126	393	393	0.83	0.61	1.12	0.20	1
Placental abruption	131	117	353	315	1.00	0.74	1.35	1	1
Placental insufficiency	209	811	728	2778	0.98	0.82	1.17	0.86	1
Preterm labor/PROM/amniotic infection	2059	6542	6675	18773	0.89	0.84	0.94	<0.001	0.001
PROM	1173	6139	3792	17336	0.87	0.81	0.94	<0.001	0.007
Pathologic CTG	525	6922	1693	19381	0.87	0.78	0.96	0.007	0.16

OR: odds ratio, CI: confidence interval; p-value from Fisher's Exact test, p-value adjustment: Holm.
PROM: premature rupture of membranes. CTG: cardiotocography.

© 2024 Staude B et al. *JAMA Network Open.*

eTable 5. Pregnancy and birth risks in 2017-2019 and 2020 in late preterm infants (32 to 36 weeks)

	**2020**		**2017 - 2019**		**odds ratio**			**Fisher exact test**	
	late preterm	term	late preterm	term	OR	lower CI	upper CI	p-value	p-adjust
Multiple pregnancy	731	723	2307	2449	1.07	0.95	1.21	0.24	1
Maternal age									
< 18 years	7	132	39	517	0.70	0.26	1.64	0.57	1
> 35 years	652	8265	2312	24849	0.85	0.77	0.93	<0.001	0.01
Previous severe maternal disease	499	5825	1701	17874	0.9	0.81	1	0.05	1
Previous caesarian section/uterine surgery	576	7866	2010	23802	0.87	0.79	0.96	0.004	0.11
Previous preterm delivery	153	780	448	2298	1.01	0.82	1.23	0.96	1
Family history of severe disease	630	8831	2234	28572	0.91	0.83	1	0.05	1
Gestational diabetes	221	2767	790	7884	0.80	0.68	0.93	0.004	0.12
Obesity	402	4920	1016	11402	0.92	0.81	1.04	0.17	1
Particular psychological burden	77	1151	385	4145	0.72	0.55	0.93	0.01	0.31
Preexisting hypertension	66	341	265	995	0.73	0.53	0.98	0.04	1
Preeclampsia/eclampsia/HELLP	222	587	831	2014	0.92	0.77	1.09	0.33	1
Bleeding < 28 weeks	42	286	127	893	1.03	0.69	1.52	0.85	1
Bleeding ≥ 28 weeks	24	46	79	148	0.98	0.53	1.77	1	1
Placenta praevia	78	126	317	393	0.77	0.55	1.07	0.11	1
Placental abruption	87	117	204	315	1.15	0.81	1.62	0.45	1
Placental insufficiency	160	811	531	2778	1.03	0.85	1.26	0.77	1
Preterm labor/PROM/amniotic infection	1665	6542	5287	18773	0.90	0.85	0.96	0.001	0.04
PROM	1029	6139	3183	17336	0.91	0.85	0.99	0.02	0.53
Pathologic CTG	421	6922	1252	19381	0.94	0.84	1.06	0.32	1

OR: odds ratio, CI: confidence interval; p-value from Fisher's Exact test, p-value adjustment: Holm.
PROM: premature rupture of membranes. CTG: cardiotocography.

© 2024 Staude B et al. *JAMA Network Open.*

eTable 6: Logistic regression models for very preterm births <32 weeks vs term born infants with covariates

	Model 0		Model Age		Model CTG		Model Indicated	
Predictors	*Odds Ratios*	*p*	*Odds Ratios*	*p*	*Odds Ratios*	*p*	*Odds Ratios*	*p*
(Intercept)	0.02	**<0.001**	0.01	**<0.001**	0.01	**<0.001**	0.01	**<0.001**
2017-19	*Reference*		*Reference*		*Reference*		*Reference*	
2020	0.87	**0.003**	0.89	**0.038**	0.87	**0.003**	0.84	**0.036**
maternal age > 35 years			1.51	**<0.001**				
2020: maternal age > 35 years			0.89	0.287				
pathologic CTG					2.03	**0.007**		
2020: pathologic CTG					1.24	0.705		
non-indicated births							11.98	**<0.001**
2020: non-indicated births							0.97	0.783
Observations	1718941		1718941		1718941		1718941	
R^2 Tjur	0.000		0.000		0.000		0.032	
AIC	27249		27182		27245		23605	

Logistic regression models were estimated using, all very preterm births (GA < 32 weeks) vs. term born infants. Covariates were maternal age >35 years (Model Age), pathologic CTG (Model CTG) and indicated vs. non-indicated births (Model Indicated). Non-indicated birth is defined as deliveries with spontaneous onset by preterm labor, premature rupture of membranes (PROM) or amniotic infection. AIC, Akaike Information Criterion.

© 2024 Staude B et al. *JAMA Network Open.*
