# Supplementary material for: Characteristics and Rates of Preterm Births During the COVID-19 Pandemic in Germany
Source: JAMA Netw Open. 2024 Sep 10;7(9):e2432438. doi: 10.1001/jamanetworkopen.2024.32438 (PMC11388025; doi:10.1001/jamanetworkopen.2024.32438)
Supplement: Supplement 2. — Data Sharing Statement [file jamanetwopen-e2432438-s002.pdf]

Data Sharing Statement

Staude. Characteristics and Rates of Preterm Births During the COVID-19 Pandemic in Germany. *JAMA Netw Open*. Published September 10, 2024.
doi:10.1001/jamanetworkopen.2024.32438

Data
Data available: No

Additional Information
Explanation for why data not available: Access to datasets of the quality assurance registry of Hesse is currently not possible for researchers. Due to the change in the quality assurance data collection instrument and data protection rules implemented on January 1st, 2021, the data cannot be made freely available within a platform for data sharing. But subcategories of the datasets can be obtained from BM upon reasonable request.
